# *Saccharomyces cerevisiae* employs complex regulation strategies to tolerate low pH stress during ethanol production

**DOI:** 10.1186/s12934-022-01974-3

**Published:** 2022-11-24

**Authors:** Yajing Wu, Bo Li, Bu Miao, Caiyun Xie, Yue-Qin Tang

**Affiliations:** 1 College of Architecture and Environment, Sichuan University, Chengdu, 610065 Sichuan China; 2 Sichuan Environmental Protection Key Laboratory of Organic Wastes Valorization, Chengdu, 610065 Sichuan China; 3 Sinopec Shanghai Engineering Co. Ltd, Shanghai, 200120 China; 4 Engineering Research Center of Alternative Energy Materials & Devices, Ministry of Education, Chengdu, 610065 Sichuan China

**Keywords:** *Saccharomyces cerevisiae*, Low pH-tolerant, Transcriptome analysis, Thiamine metabolism, Ethanol production

## Abstract

**Background:**

Industrial bioethanol production may involve a low pH environment caused by inorganic acids, improving the tolerance of *Saccharomyces cerevisiae* to a low pH environment is of industrial importance to increase ethanol yield, control bacterial contamination, and reduce production cost. In our previous study, acid tolerance of a diploid industrial *Saccharomyces cerevisiae* strain KF-7 was chronically acclimatized by continuous ethanol fermentation under gradually increasing low-pH stress conditions. Two haploid strains B3 and C3 having excellent low pH tolerance were derived through the sporulation of an isolated mutant. Diploid strain BC3 was obtained by mating these two haploids. In this study, B3, C3, BC3, and the original strain KF-7 were subjected to comparison transcriptome analysis to investigate the molecular mechanism of the enhanced phenotype.

**Result:**

The comparison transcriptome analysis results suggested that the upregulated vitamin B1 and B6 biosynthesis contributed to the low pH tolerance. Amino acid metabolism, DNA repairment, and general stress response might also alleviate low pH stress.

**Conclusion:**

*Saccharomyces cerevisiae* seems to employ complex regulation strategies to tolerate low pH during ethanol production. The findings provide guides for the construction of low pH-tolerant industrial strains that can be used in industrial fermentation processes.

**Supplementary Information:**

The online version contains supplementary material available at 10.1186/s12934-022-01974-3.

## Background

As a renewable clean energy, bioethanol is an important substitute for gasoline. The research on bioethanol production has an important social, economic, and environmental significance. *Saccharomyces cerevisiae*, the main species used in industrial bioethanol production, grows optimally in pH 4.0–6.0 conditions [1]. In industrial bioethanol production using cell cycle technology, inorganic acid such as sulfuric acid is often used to clean yeast cells to control bacterial contamination [2–4]. In addition, lowering the pH is a general method to reduce the risk of bacterial contamination in bioethanol fermentation [4, 5]. In recent years, lignocellulosic biomass has been widely used in bioethanol production. The pretreatment of lignocellulosic biomass may involve harsh inorganic acid pre-treatment which results in a relatively low pH of pretreated material [4, 6]. The extensive washing and/or detoxification to remove the acid before fermentation will inevitably increase the cost. Meanwhile, low pH may reduce cell viability and consequently the fermentation yield [2, 4, 7]. Therefore, improving the tolerance of *S. cerevisiae* to a low pH environment caused by inorganic acids may be of industrial importance to control bacterial contamination, increase ethanol yield and reduce production cost.

Current researches mostly adopt traditional methods to breed low pH-tolerant strains, such as continuous acclimation under low pH conditions, haploidization, and mating. Fletcher et al. obtained an HCl-tolerant yeast HCl-1 from haploid *S. cerevisiae* CEN.PK 113-7D by continuous transfer for 281 generations under gradually decreasing pH conditions [4]. Mitsumasu et al. obtained two diploid mutant strains by continuous fermentation under acidic or high-temperature conditions, followed by haploidization and mating, and strain AT with significantly improved low pH- and thermo-tolerance was developed [8]. Benjaphokee et al. obtained *S. cerevisiae* TJ14 with improved heat and acid tolerance by spore-to-cell mating of strains with different phenotypes [9]. These traditional breeding methods are time-consuming and laborious. However, the molecular mechanism of yeast's long-term tolerance to low pH is still unclear. To date, only few studies regulated individual genes through rational genetic engineering to improve the low pH tolerance of *S. cerevisiae*. For example, Matsushika et al. found that *GAS1* overexpression can improve the low pH tolerance of *S. cerevisiae* [10]. Therefore, systematic elucidation of the low pH tolerance mechanism of *S. cerevisiae* would provide potential modifying targets for the construction of low pH-tolerant strains by genetic engineering. It has a guiding significance for many industrial fermentation processes, besides ethanol production.

Studies have shown that low pH activates general stress response (GSR) pathway [3, 7, 11]. Protein kinase A (PKA) and protein kinase C (PKC) pathway-dependent regulatory mechanisms may affect low pH tolerance by affecting cell cycle, cell wall integrity (CWI), etc. [3, 7, 11]. Genes related to CWI, high osmolarity glycerol (HOG) pathway, redox processes, carbohydrate metabolism, ATP synthesis, ion uptake, etc., are critical for cell survival and low-pH stress adaptation [7, 11]. Although the relevant research is very limited, it has been found that strains with different genetic backgrounds have different responses when exposed to low pH condition. For strain BY4741, low pH also caused coordinated downregulation of genes involved in ion uptake, the de novo synthesis of purines was repressed [7], and the Ca^2+^ calmodulin-dependent calcineurin pathway participated in low pH tolerance [11]. By using the BY4741 knockout library, it was found that the expression of genes involved in ion homeostasis, cell surface genes, nutrient transporters, vacuolar, cell surface networks, endosomal, and vesicle-mediated transport processes responded strongly to strong acids of HCl and H_2_SO_4_ [1]. Mutant strain HCl-1 possibly achieved low pH tolerance by changing sterol composition and regulating intracellular iron levels [4].

As the studies on the molecular mechanism to low pH tolerance of *S. cerevisiae* were limited and the different genetic backgrounds of different strains have a significant impact on the tolerance mechanism, it is, therefore, necessary to carry out more systematic and rigorous research and accumulate relevant research data using industrial strains. It is very important for establishing a universal molecular regulation strategy. In our previous study, using diploid industrial *S. cerevisiae* strain KF-7 [12] as the original strain, a mutant strain was obtained by long-term continuous ethanol fermentation under gradually increasing low-pH stress conditions. The mutant strain was haploidized, and two haploid strains B3 (*MAT*a) and C3 (*MAT*α) having excellent low pH tolerance were obtained, then a diploid strain BC3 was obtained by mating B3 and C3 [13]. In this study, by gene microarray analysis, the gene expression differences between the original strain and three low pH-tolerant strains under the conditions of pH 4.5 and pH 2.5, respectively, were compared. By analyzing the differentially expressed genes (DEGs) unique under pH 2.5 condition, the molecular mechanism of low pH tolerance was described more accurately.

## Results

### Batch fermentation

**Fig. 1:**
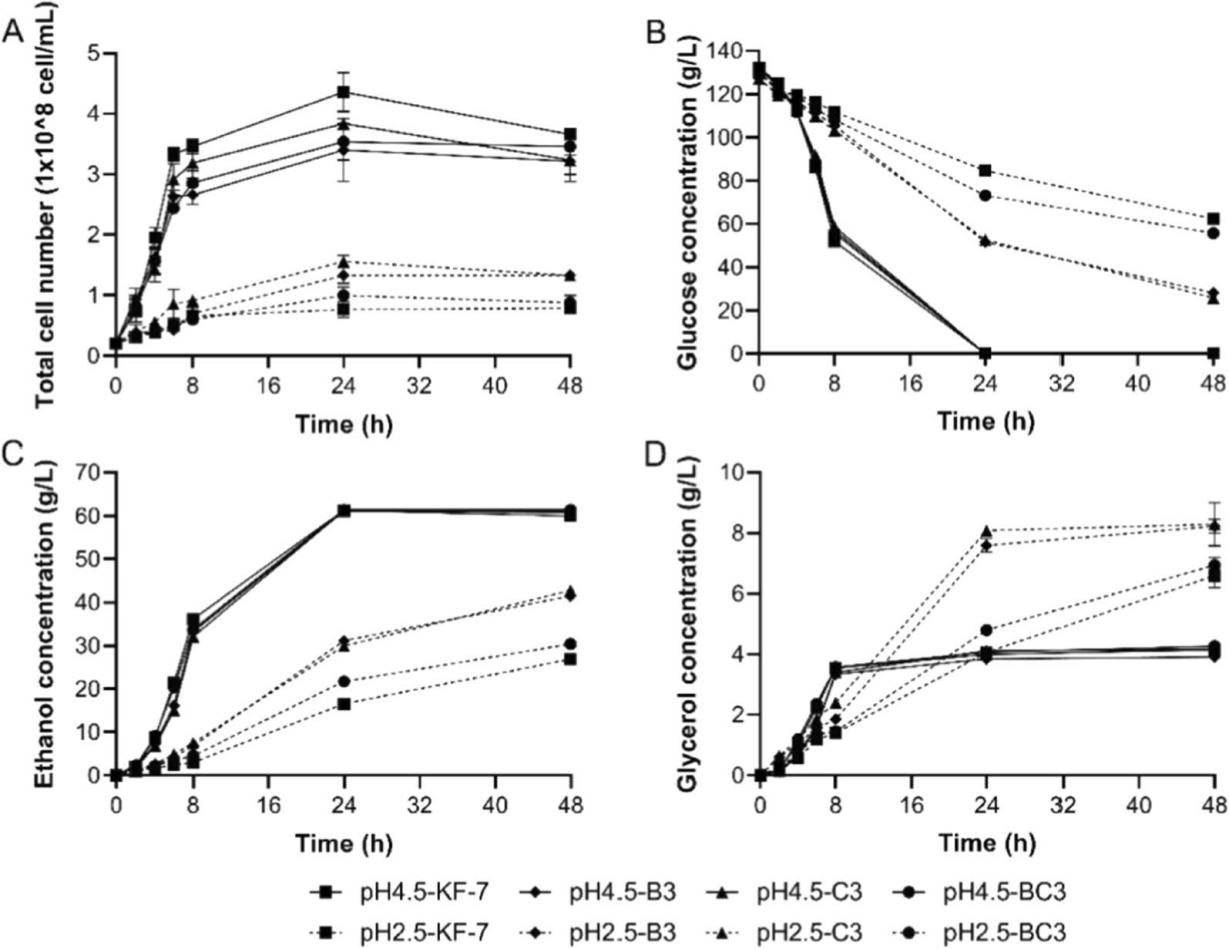
Batch fermentation of KF-7 (square), B3 (diamond), C3 (triangle), and BC3 (circle) in the conditions of pH 4.5 (solid line) and pH 2.5 (dotted line). Total cell number (**A**) and the concentrations of glucose (**B**), ethanol (**C**), and glycerol (**D**) were measured. The average values and standard derivations (error bars) of three independent experiments were presented

Batch fermentations were carried out with YPD130 medium under pH 2.5 and pH 4.5, respectively. As shown in Fig. 1, under pH 4.5, the growth of the original strain KF-7 was the fastest. Glucose consumption rates of strains were similar, and the glucose was completely consumed within 24 h. After 48 h of fermentation, the ethanol concentration of each strain reached about 60 g/L, and the ethanol yields based on the consumption of glucose of KF-7, B3, C3, and BC3 were 88.98%, 90.79%, 90.36%, and 91.01%, respectively. The glycerol concentrations and yields based on the consumption of glucose (about 0.03 g/g) of the four strains were also similar.

Under pH 2.5, the growth and fermentation capacity of each strain was inhibited to varying degrees (Fig. 1). The growth rate of haploid B3 and C3 was higher than those of BC3 and KF-7, while the glucose consumption rate and ethanol concentration were significantly higher than those of BC3 and KF-7. After 24 h of fermentation, the glucose consumption rates of KF-7, B3, C3, and BC3 were 1.90, 3.16, 3.11, and 2.38 g/(L·h), and the ethanol production rates were 0.69, 1.30, 1.25 and 0.91 g/(L·h), respectively. After 48 h of fermentation, the remaining glucose concentrations were 62.38, 28.15, 25.86, and 55.71 g/L, respectively. The ethanol concentrations were 26.95, 41.50, 42.77, and 30.44 g/L, and the ethanol yields based on glucose consumption were 77.72%, 82.06%, 82.64%, and 79.96%, respectively. Haploid strains B3 and C3 had higher glycerol concentrations than KF-7 and BC3, but slightly lower glycerol yields based on glucose consumption (0.083, 0.082 g/g) than KF-7 and BC3 (0.097, 0.093 g/g).

At present, the reports on the fermentation capacity of *S. cerevisiae* under low pH of 2.5 is limited. Under the conditions of pH 2.5 and initial glucose concentration close to the present study, the glucose consumption rate of *S. cerevisiae* ITV-01 [14] was significantly lower than that of B3 and C3. The amount of glucose consumed during 36 h-fermentation is equivalent to those of B3 and C3 during 24 h-fermentation, and the ethanol yield of ITV-01was also lower than those of B3 and C3.

### Transcriptome analysis

The transcriptome results of the 16 RNA samples were shown in Additional file 1: Fig. S1. Parallel samples showed good repeatability. The transcription levels of *ADY2*, *ATO2*, *BTN2*, *ENO1*, *ENO2,* and *HSP30* of all 16 RNA samples were analyzed by RT-qPCR. The results of RT-qPCR were consistent with the results of the transcriptome analysis, suggesting that the transcriptomic results were reliable (Additional file 1: Fig. S2).

**Fig. 2:**
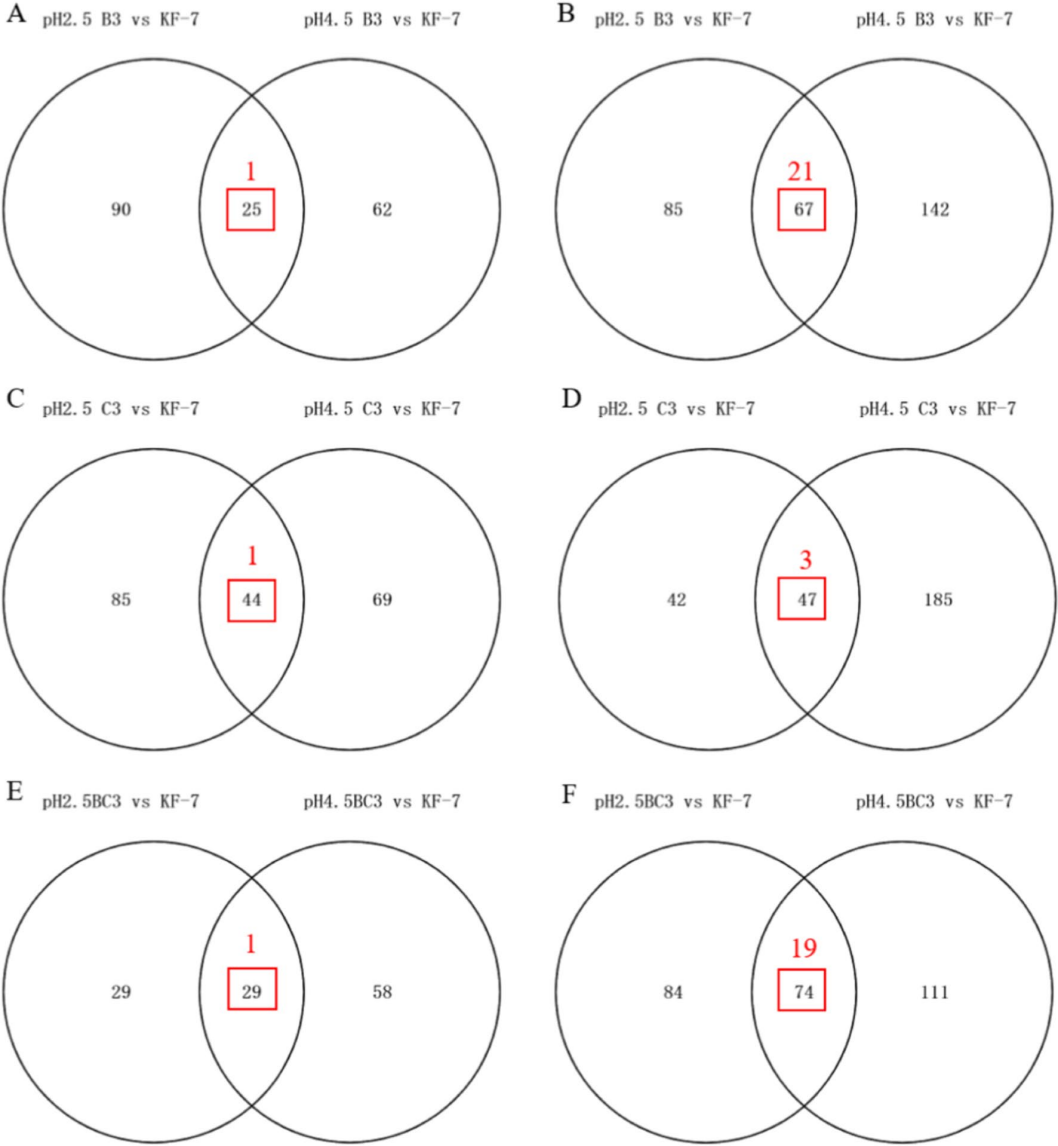
Venn diagram of DEGs between the conditions of pH 2.5 and pH 4.5 in groups B3 vs KF-7 **A**, **B**, C3 vs KF-7 **C**, **D**, and BC3 vs KF-7 **E**, **F**. **A**, **C**, and **E** indicated the significantly upregulated genes while **B**, **D**, and **F** indicated the significantly downregulated genes. The red numbers were the numbers of DEGs whose fold change at pH 2.5/ fold change at pH 4.5 > 1.5 (for upregulated DEGs shared by both pH conditions) or < 0.67 (for downregulated DEGs shared by both pH conditions)

Taking the original strain KF-7 as the control, the number of DEGs of each low pH-tolerant strain under the two pH conditions were shown in Fig. 2. Under pH 4.5, the numbers of DEGs of B3, C3, and BC3 were 296 (upregulation 87, downregulation 209), 345 (upregulation 113, downregulation 232), and 272 (upregulation 87, downregulation 185), respectively. Under pH 2.5, the numbers of DEGs of B3, C3, and BC3 were 267 (upregulation 115, downregulation 152), 218 (upregulation 129, downregulation 89), and 216 (upregulation 58, downregulation 158), respectively.

In this study, only the DEGs unique under pH 2.5 condition and the DEGs whose fold change at pH 2.5/ fold change at pH 4.5 > 1.5 (for upregulated DEGs shared by both pH conditions) or < 0.67 (for downregulated DEGs shared by both pH conditions) were used for subsequent analysis. The numbers of DEGs for subsequent analysis were as follows: those unique under pH 2.5 condition were 171 (upregulation 90, downregulation 85), 125 (upregulation 85, downregulation 42), 112 (upregulation 29, downregulation 84), and those shared by both pH conditions were 22 (upregulation 1, downregulation 21), 4 (upregulation 1, downregulation 3), 20 (upregulation 1, downregulation 19) for strains B3, C3, and BC3, respectively (Fig. 2).

**Fig. 3:**
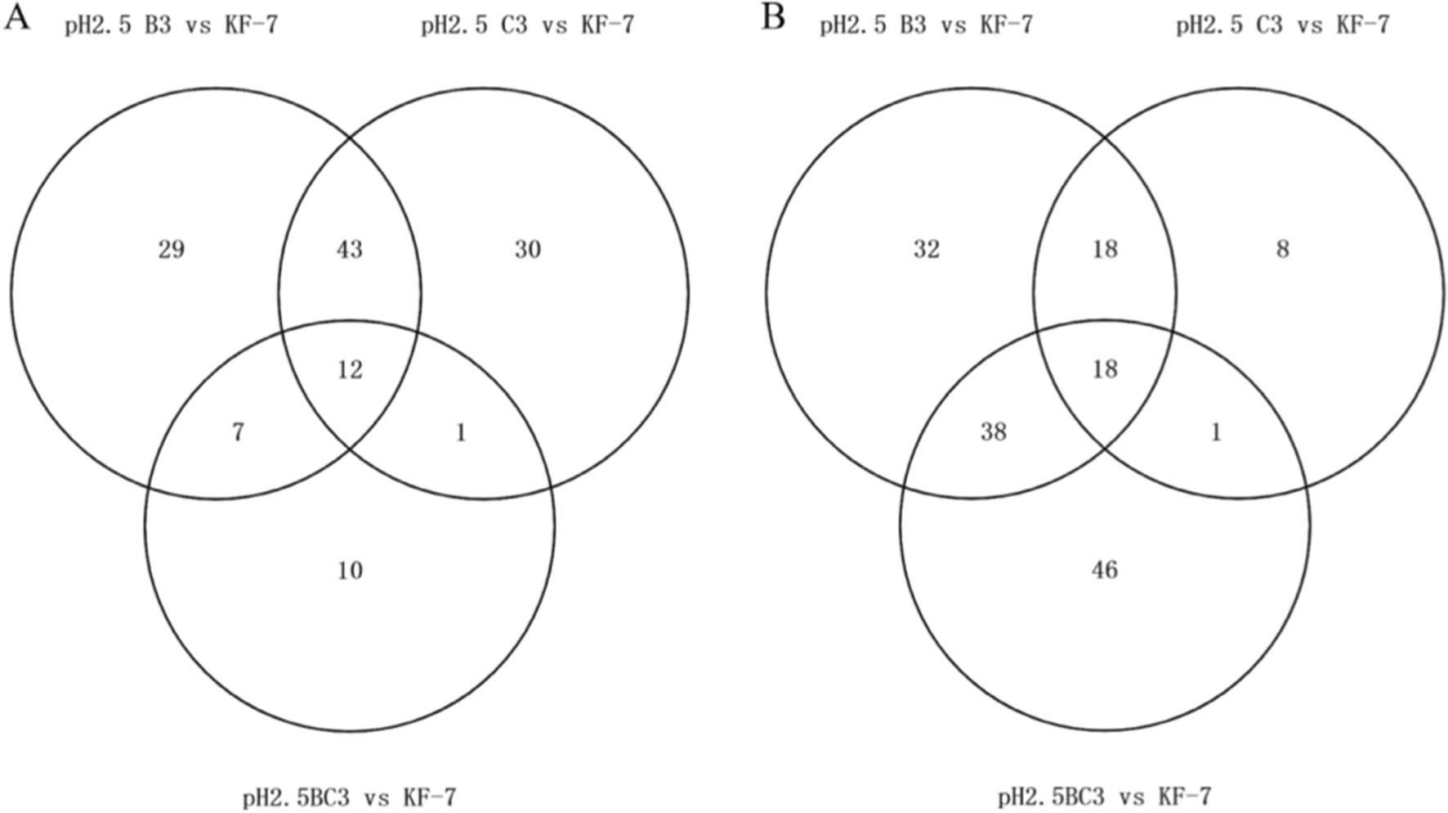
Venn diagram of the upregulated **A** and downregulated **B** DEGs in groups B3 vs KF-7, C3 vs KF-7, and BC3 vs KF-7 at pH 2.5

Among the upregulated DEGs, there were 55 DEGs shared by B3 and C3, and only 12 of these 55 DEGs were shared by BC3 (Fig. 3). Among the downregulated DEGs, there were 36 DEGs shared by B3 and C3, and only 18 of these 36 DEGs were shared by BC3. The type of DEGs of BC3 was significantly different from that of B3 and C3, indicating that the response of BC3 to pH 2.5 was significantly different from that of B3 and C3. This might be the reason why the low pH tolerance of BC3 was much lower than that of B3 and C3.

### Analysis of DEGs involved in low pH tolerance

**Fig. 4:**
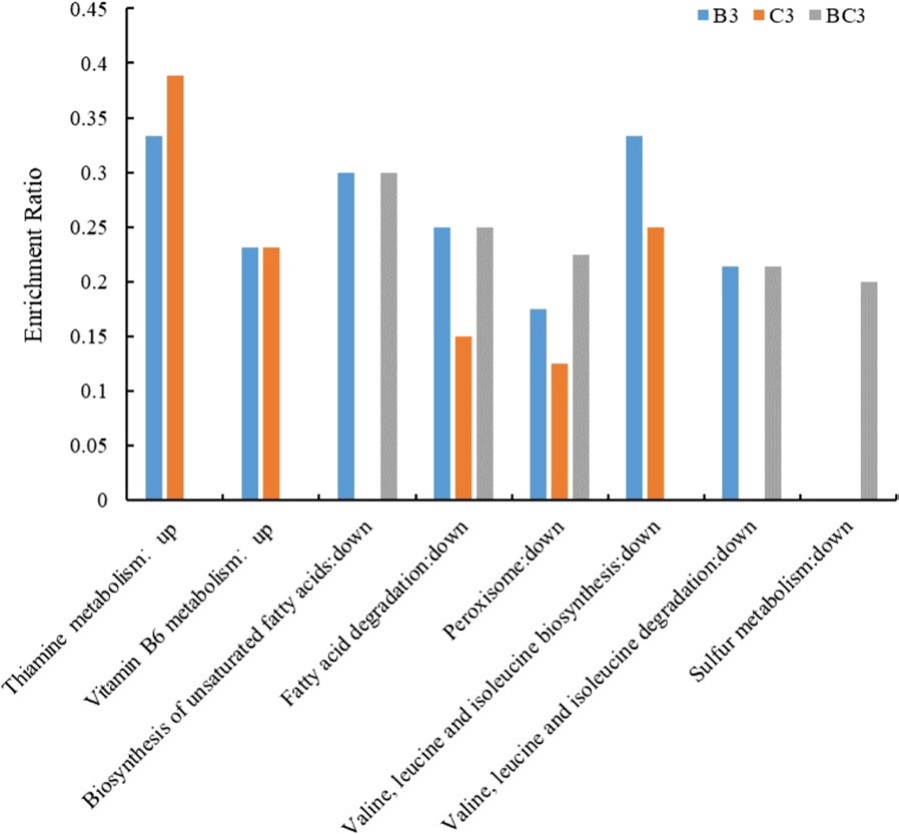
The significantly enriched KEGG pathway at pH 2.5 (*P* ≤ 0.05). The enrichment ratio of each KEGG term was the number of DEGs involved in each KEGG term to the number of total genes involved in each KEGG term. B3, C3, and BC3 indicated groups B3 vs KF-7, C3 vs KF-7, and BC3 vs KF-7, respectively

The Kyoto Encyclopedia of Genes and Genomes (KEGG) pathways enriched for DEGs of B3, C3, and BC3 at pH 2.5 were shown in Fig. 4. Thiamine (vitamin B1) and vitamin B6 metabolism were enriched for the upregulated DEGs of B3 and C3, fatty acid metabolism and amino acid metabolism were enriched for the downregulated DEGs. No pathway was enriched for upregulated DEGs of BC3, while fatty acid metabolism, amino acid metabolism, and sulfur metabolism were enriched for downregulated DEGs of BC3. Fatty acid metabolism and amino acid metabolism, the common KEGG pathways enriched for all three strains, might contribute to the improved low pH tolerance. Thiamine and vitamin B6 metabolism, the enriched KEGG pathway unique for B3 and C3, might contribute to the better low pH tolerance of B3 and C3, compared to BC3.

#### Fatty acid metabolism

Fatty acid metabolism was enriched for B3, C3, and BC3. As shown in Table 1, most DEGs were downregulated, and the downregulation degrees of B3 and BC3 were slightly higher than that of C3. In yeast cells, fatty acid degradation exclusively occurs in peroxisomes. Fatty acids can be degraded by peroxisomal beta-oxidation to produce acetyl-CoA [15, 16].

**Table 1:** Fold changes of DEGs involved in the fatty acid degradation pathway

DEGs	Function	B3 vs KF-7	C3 vs KF-7	BC3 vs KF-7	Notes
*POX1*	acyl-CoA oxidase	0.13*	**0.20**	**0.11**	Enzymes involved in β-oxidation [17]
		0.52**	**0.33**	0.53	
*POT1*	β-ketoacyl-CoA thiolase	**0.06**	**0.11**	**0.05**	
		**0.17**	**0.15**	**0.23**	
*SPS19*	2,4-dienoyl-CoA reductase	**0.42**	0.51	**0.31**	
		0.83	0.70	0.69	
*ECI1*	Δ^3^-Δ^2^-enoyl-CoA isomerase	**0.36**	**0.44**	**0.37**	
		0.68	0.60	0.67	
*DCI1*	Δ^3;5^-Δ^2;4^-dienoyl CoA isomerase	0.52	0.67	**0.49**	
		0.83	1.03	0.73	
*CAT2*	carnitine acetyltransferase	0.53	0.63	**0.49**	Other enzymes involved in fatty acid breakdown [17]
		0.65	0.77	0.66	
*CTA1*	catalase A	**0.34**	**0.34**	**0.34**	
		0.72	0.55	0.50	
*IDP2*	isocitrate dehydrogenase	**0.28**	**0.37**	**0.28**	
		**0.41**	0.52	**0.42**	
*TES1*	acyl-CoA thioesterase	**0.42**	0.55	**0.49**	
		0.60	0.53	0.54	
*ADR1*	transport factor	**0.19**	**0.34**	**0.22**	
		**0.42**	**0.35**	0.51	

Bold type indicated downregulation

^*^Data in the first row were derived from pH 2.5

^**^Data in the second row were derived from pH 4.5

#### Thiamine and vitamin B6 biosynthesis

**Fig. 5:**
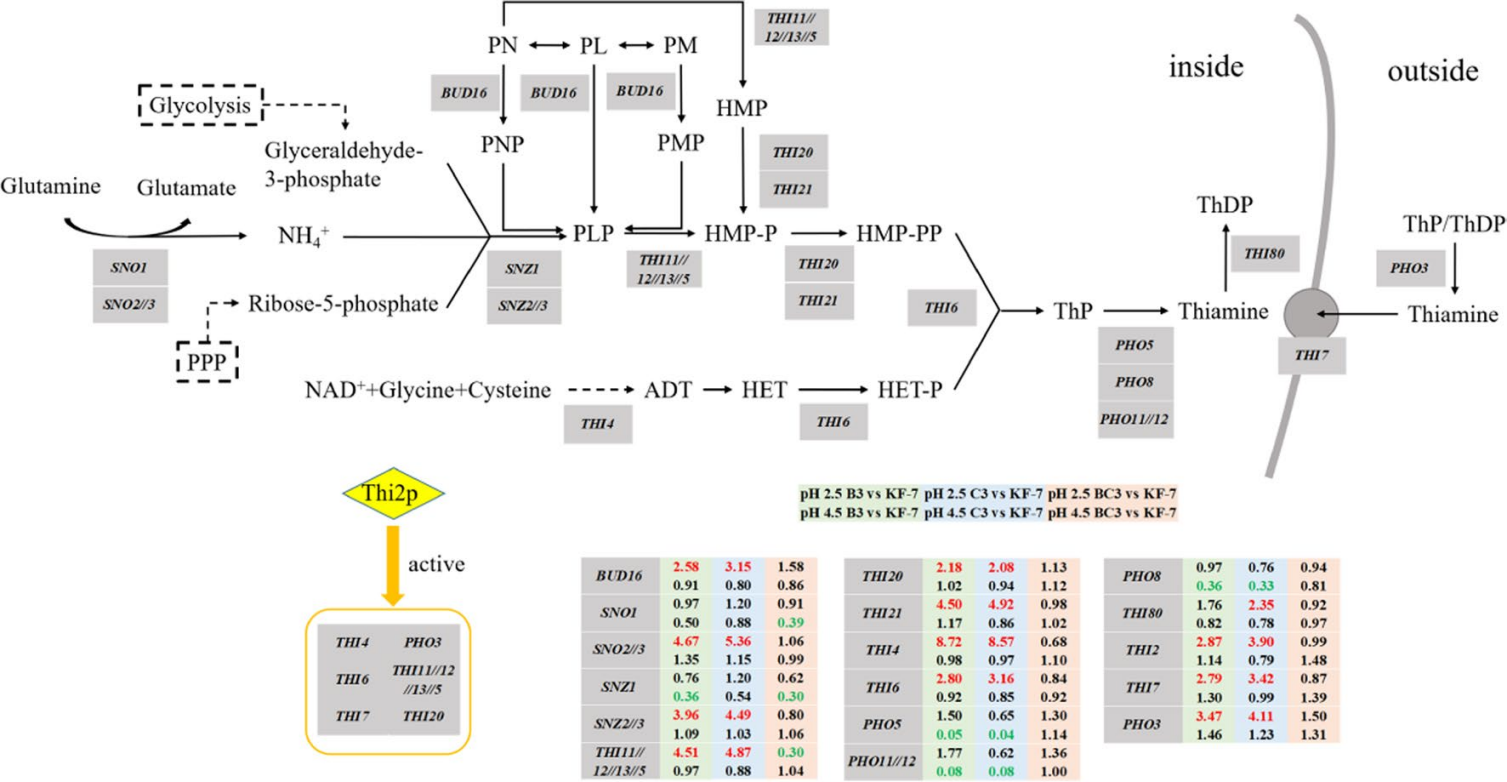
DEGs involved in thiamine and vitamin B6 biosynthesis pathway. Green, blue, and red backgrounds indicated groups B3 vs KF-7, C3 vs KF-7, and BC3 vs KF-7, respectively. Letters in red indicated upregulation and in green indicated downregulation. Data in the first row were derived from pH 2.5, and data in the second row were derived from pH 4.5

At pH 2.5, the most significant difference between haploids B3, C3, and diploid BC3 was the biosynthesis of thiamine (vitamin B1) and vitamin B6. The DEGs involved in thiamine and vitamin B6 biosynthesis, and their expressions were shown in Fig. 5. There are 35 genes involved in the biosynthesis of thiamine and thiamine diphosphate (ThDP) from vitamin B6 in *S. cerevisiae*, 15 of them were upregulated in B3 and C3, including *BUD16*, *SNO2*//*3*, *SNZ2*//*3*, *THI11*//*12*//*13*//*5*, *THI20*, *THI21*, *THI4*, *THI6*, *PHO3*, and *THI80*.

Vitamin B6 is a generic term describing a group of three water-soluble vitamins, pyridoxine (PN), pyridoxal (PL), pyridoxamine (PM), and the 5′-phosphates derived therefrom [18, 19]. They are intermediates in thiamine synthesis [19]. The pyridoxal 5′-phosphate (PLP) is the biologically active form of vitamin B6 and has multiple roles as a versatile cofactor of enzymes [18, 20]. *BUD16* encodes yeast pyridoxal kinase (Pdxk), which is a critical enzyme in PLP metabolism [20]. *S. cerevisiae* contains three paralogs of the two-component *SNZ*/*SNO* PLP synthase, *SNZ1*//*2*//*3* encoding PLP synthase while *SNO1*//*2*//*3* encoding glutaminase enzymes [21]. The transcriptional profile was different between *SNZ1*/*SNO1* and *SNZ2*//*3*/*SNO2*//*3*. This might be due to the thiamine depletion and the growth stage. The expression of *SNZ2*//*3* and *SNO2*//*3* genes could be induced by the absence of thiamine, rather than those of *SNZ1* and *SNO1* [22]. *SNO1* and *SNZ1* are more expressed during the diauxic shift and/or stationary phase of growth, while *SNO2*//*3* and *SNZ2*//*3* are more expressed at the logarithmic phase [22, 23]. According to growth evaluation (Fig. 1A), the cells were in logarithmic phase when yeast cells used for transcriptome analysis were harvested at 4 h under pH 4.5 and 8 h under pH 2.5. The increased expression of *SNO2*//*3* and *SNZ2*//*3* genes in B3 and C3 might be the result of gene amplification in genome, since there are several studies reported that these genes were amplified in industrial fuel-ethanol yeast strains capable of efficient fermentation of sugar with high concentrations [24–26].

Thiamine has a two-ring structure: 4-amino-2-methyl-5-hydroxymethylpyrimidine (HMP) and 4-methyl-5-β-hydroxyethylthiazole (HET), connected through a methylene bridge [27, 28]. The *THI5* family (*THI5*, *THI11*, *THI12*, and *THI13*) catalyzes the reaction from PN to HMP [29], and that from PLP to HMP-P [21]. *THI20* and *THI21* encode HMP kinase and HMP-P kinase [30]. *THI4* encodes the thiazole synthase required for HET synthesis [29]. The enzyme encoded by *THI6* has both the function of pyrophosphorylation of thiamine phosphate and the function of phosphorylation of HET [28]. Condensation of the phosphorylated HET and HMP to generate thiamin phosphate (ThP) occurs via thiamin phosphate pyrophosphorylase [28]. *THI80* encodes thiamine pyrophosphokinase which catalyzes the final step of the thiamine synthesis pathway. ThDP, the active form of thiamine, is synthesized by this enzyme which is essential in *S. cerevisiae* [28].

Thi2p functions as a positive regulator of some *THI* genes [30, 31]. *THI7* and *PHO3* are involved in the acquisition of exogenously available thiamin [31]. *THI7* encodes a plasma membrane thiamine transporter [30–32]. *PHO3* encodes a periplasmic acid phosphatase that catalyzes the release of thiamine from thiamine phosphates [28, 30].

#### Amino acid metabolism

**Fig. 6:**
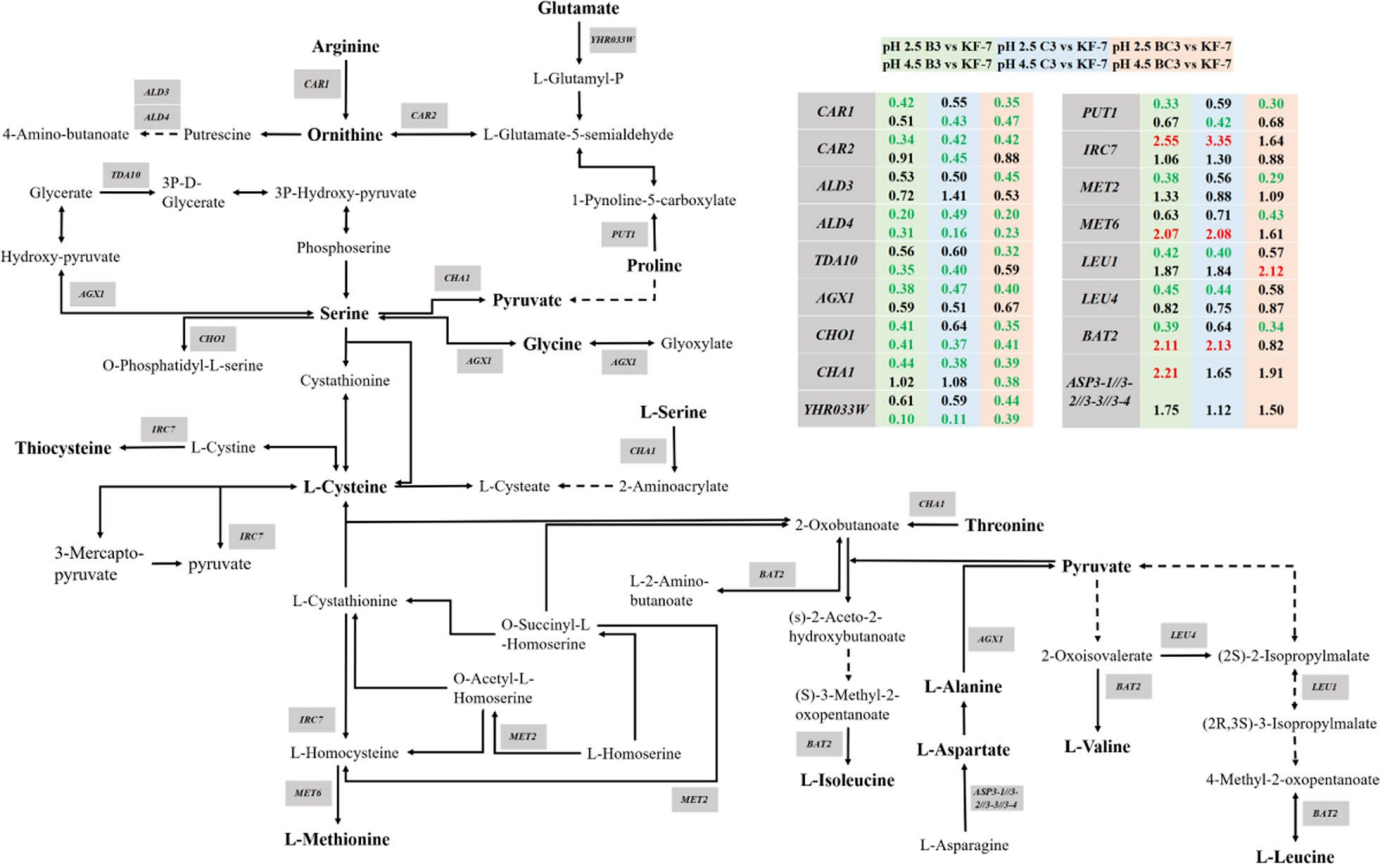
DEGs involved in amino acid metabolism. Green, blue, and red backgrounds indicated groups B3 vs KF-7, C3 vs KF-7, and BC3 vs KF-7, respectively. Letters in red indicated upregulation and in green indicated downregulation. Data in the first row were derived from pH 2.5, and data in the second row were derived from pH 4.5

Amino acid metabolism pathways were enriched for the DEGs of B3, C3, and BC3. Most of the DEGs in these pathways were downregulated, including those in leucine synthesis (*LEU1*, *LEU4*, *BAT2* downregulation), methionine synthesis (*MET2*, *MET6* downregulation), glycine synthesis (*AGX1* downregulation), aspartate synthesis (*ASP3* upregulation), proline degradation (*PUT1* downregulation), serine degradation (*CHO1*, *CHA1* downregulation), and arginine degradation (*CAR1*, *CAR2* downregulation) (Fig. 6).

#### Central carbon metabolism

**Fig. 7:**
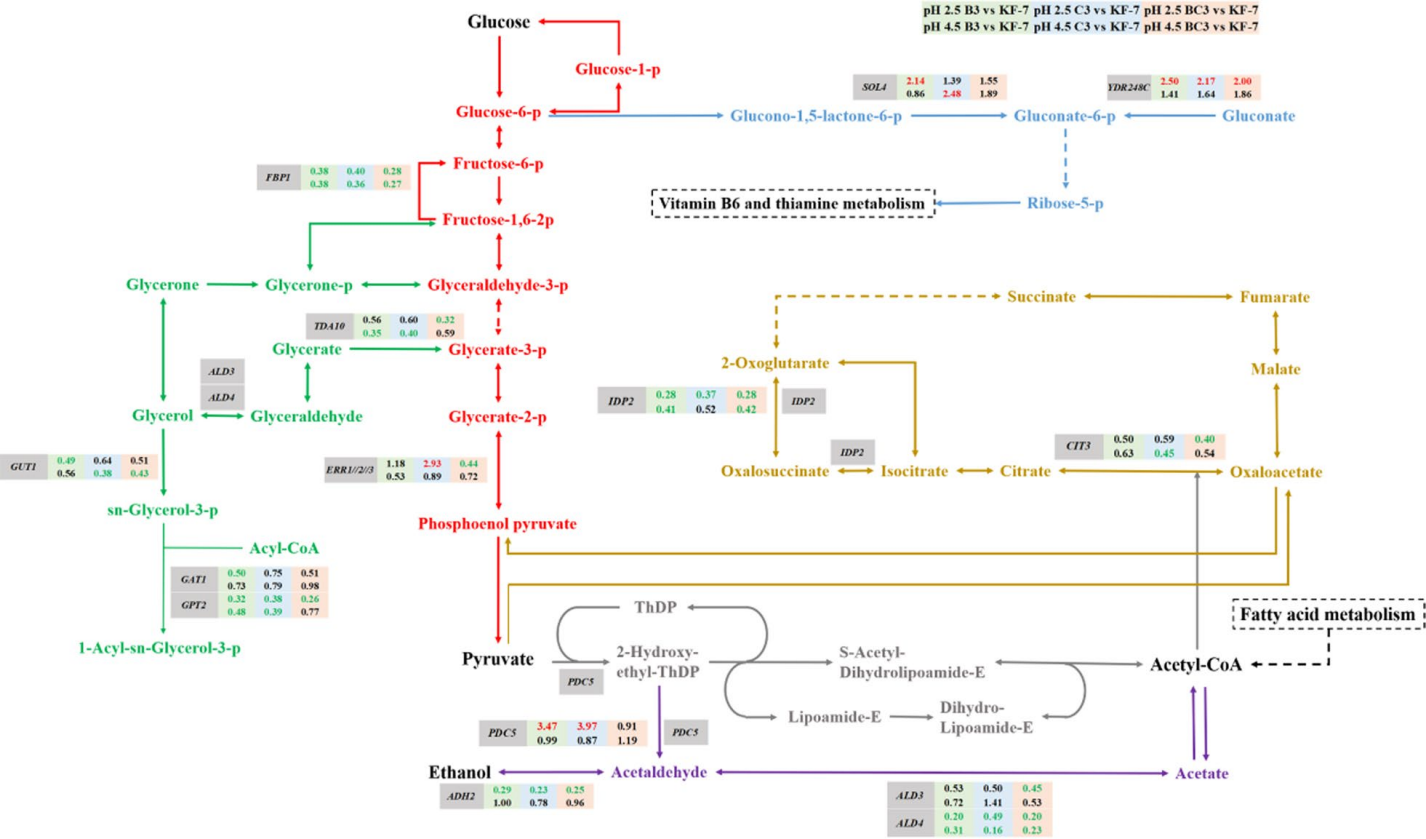
DEGs involved in the central carbon metabolism. Green, blue, and red backgrounds indicated groups B3 vs KF-7, C3 vs KF-7, and BC3 vs KF-7, respectively. Letters in red indicated upregulation and in green indicated downregulation. Data in the first row were derived from pH 2.5, and data in the second row were derived from pH 4.5

Under pH 2.5, the glucose metabolism rate and ethanol production rate of the original strain and the low pH-tolerant strains were significantly different. The DEGs involved in central carbon metabolism were analyzed (Fig. 7). Limited genes involved in central carbon metabolism were differentially expressed, including *FBP1*, *ERR1*//*2*//*3*, *SOL4*, *YDR248C*, *IDP2*, *CIT3*, *PDC5*, *ADH2*, *ALD3,* and *ALD4*.

The ethanol yields of B3, C3, and BC3 were all higher than that of the original strain KF-7, probably due to the upregulation of *PDC5* and the downregulation of *ALD3* and *ADH2*. *PDC5* encodes pyruvate decarboxylase which converts pyruvate to acetaldehyde. *ALD3* encodes acetaldehyde dehydrogenase which converts acetaldehyde to acetate. *ADH2* encodes alcohol dehydrogenase which converts ethanol to acetaldehyde. The differential expression of those genes might enhance the conversion of acetaldehyde to ethanol. Although the glycerol yield of each low pH-tolerant strain increased at pH 2.5 when compared with that at pH 4.5, the DEGs involved in glycerol synthesis were not upregulated. However, *GUT1*, *GAT1,* and *GPT2*, the genes participating in glycerol degradation were downregulated and the fold changes of these genes were similar in B3, C3, and BC3.

#### General stress response (GSR)

Although the KEGG pathway of GSR was not enriched for DEGs at pH 2.5, previous studies found that there was a great correlation between thiamine, amino acid, glycerol and GSR. In addition, it has been reported that yeast cells respond promptly to pH reduction by inducing the expression of genes involved in GSR [3, 11]. Therefore, we also focused on DEGs involved in GSR under pH 2.5.

Under pH 2.5, the major DEGs related to stress response were shown in Table 2. The DEGs were mainly *HSP* genes, which were upregulated except for *HSP12*. Other DEGs involved in stress response mainly include *SMP1*, *MSN4*, *ZNF1*, *PDR12*, and *PDR15*. *SMP1* is involved in cellular osmotic stress tolerance, and *MSN4* can respond to multiple stresses. The functions of Smp1p and Msn4p were reported related to the Hog1-MAPK (Mitogen-activated protein kinase) pathway [33]. *ZNF1* played a role in tolerance to pH and osmotic stress [34]. *PDR12* was reported related to weak acid tolerance [35].

**Table 2:** Major DEGs involved in stress responses

DEGs	B3 vs KF-7	C3 vs KF-7	BC3 vs KF-7	Notes
*HSP32*//*33*//*SNO4*	1.45*	2.08^a^	0.91	Coding heat-shock proteins
	0.66**	1.12	0.99	
*HSP12*	0.46^b^	0.78	0.36^b^	
	0.61	0.93	0.85	
*HSP26*	2.56^a^	2.81^a^	0.87	
	0.58	0.96	1.80	
*HSP30*	3.23^a^	2.00	2.97^a^	
	1.41	3.46^a^	3.51^a^	
*SMP1*	3.53^a^	2.78^a^	3.14^a^	Others
	0.86	0.81	0.81	
*ZNF1*	0.38^b^	0.40^b^	0.34^b^	
	0.56	0.39^b^	0.30^b^	
*MSN4*	0.64	0.95	0.49^b^	
	0.36^b^	0.34^b^	0.62	
*PDR12*	0.47^b^	0.39^b^	0.95	
	1.35	0.94	1.33	
*PDR15*	0.19^b^	0.33^b^	0.19^b^	
	1.55	1.21	2.28^a^	

^*^Data in the first row were derived from pH 2.5

^**^Data in the second row were derived from pH 4.5

^a^upregulation, ^b^downregulation

#### DNA repairment

Low pH may adversely affect DNA replication, transcription, translation, or DNA stability. We found that many DEGs related to DNA repairment were upregulated in B3 and C3, while fewer of them were upregulated in BC3 (Table 3).

**Table 3:** Fold changes of major upregulated DEGs involved in DNA repairment

DEGs	B3 vs KF-7	C3 vs KF-7	BC3 vs KF-7
*BUD16*	2.58*^a^	3.15^a^	1.58
	0.91**	0.80	0.86
*THI4*	8.72^a^	8.57^a^	0.68
	0.98	0.97	1.10
*YJR096W*	2.54^a^	2.19^a^	1.72
	0.66	1.36	1.14
*YGR126W*	1.86	2.15^a^	1.57
	1.13	1.99	1.18
*MAC1*	1.97	2.20^a^	1.57
	0.62	0.77	0.72
*YLR118C*	2.33^a^	2.32^a^	1.54
	1.03	1.16	1.09
*RAD10*	2.24^a^	2.09^a^	1.88
	0.92	1.08	0.93
*MND1*	1.84	2.18^a^	1.45
	0.72	0.59	0.91
*PHR1*	2.51^a^	2.35^a^	1.94
	0.56	0.59	0.60
*HST4*	2.11^a^	2.72^a^	1.76
	0.56	0.71	0.58

^*^Data in the first row were derived from pH 2.5

^**^Data in the second row were derived from pH 4.5

^a^upregulation

#### Protein interaction analysis of DEGs of B3 and C3

**Fig. 8:**
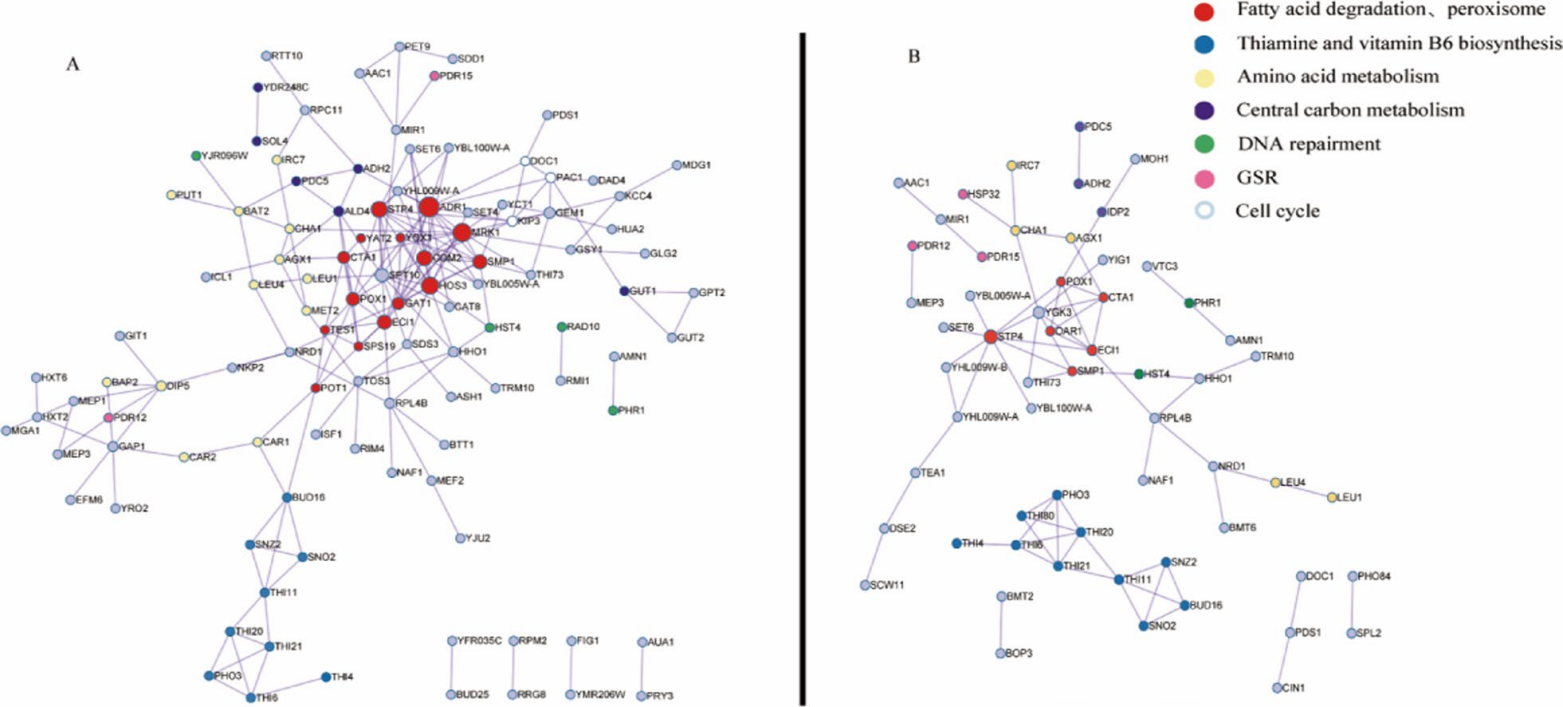
Protein interaction analysis of DEGs in groups B3 vs KF-7 **A** and C3 vs KF-7 **B** at pH 2.5

The association of DEGs of B3 and C3 at pH 2.5 was explored by protein interaction analysis to further understand the mechanism of low pH tolerance of *S. cerevisiae*. As shown in Fig. 8, the DEGs involved in fatty acid metabolism were at the core of the network. The DEGs *MRK1*, *HOS3*, *SMP1*, *COM2*, and *GAT1*, closely associated with DEGs of fatty acid metabolism, might play important roles in the low pH tolerance, although these DEGs were not included in enriched KEGG pathways. The DEGs involved in thiamine and vitamin B6 metabolism were grouped in a relatively independent position and the group was associated with other DEGs through *CAR1* and *POT1*. The positions of DEGs involved in amino acid metabolism, central carbon metabolism, DNA repair, GSR, and cell cycle were scattered in the network. Although no pathway was enriched for DEGs such as *SPT10*, *YGK3*, *GEM1*, and *HOO1*, these DEGs were closely associated with other DEGs. The regulatory mechanisms of these DEGs and their relationships remain to be further explored.

## Discussion

### Maintenance of cellular homeostasis under low pH condition

**Fig. 9:**
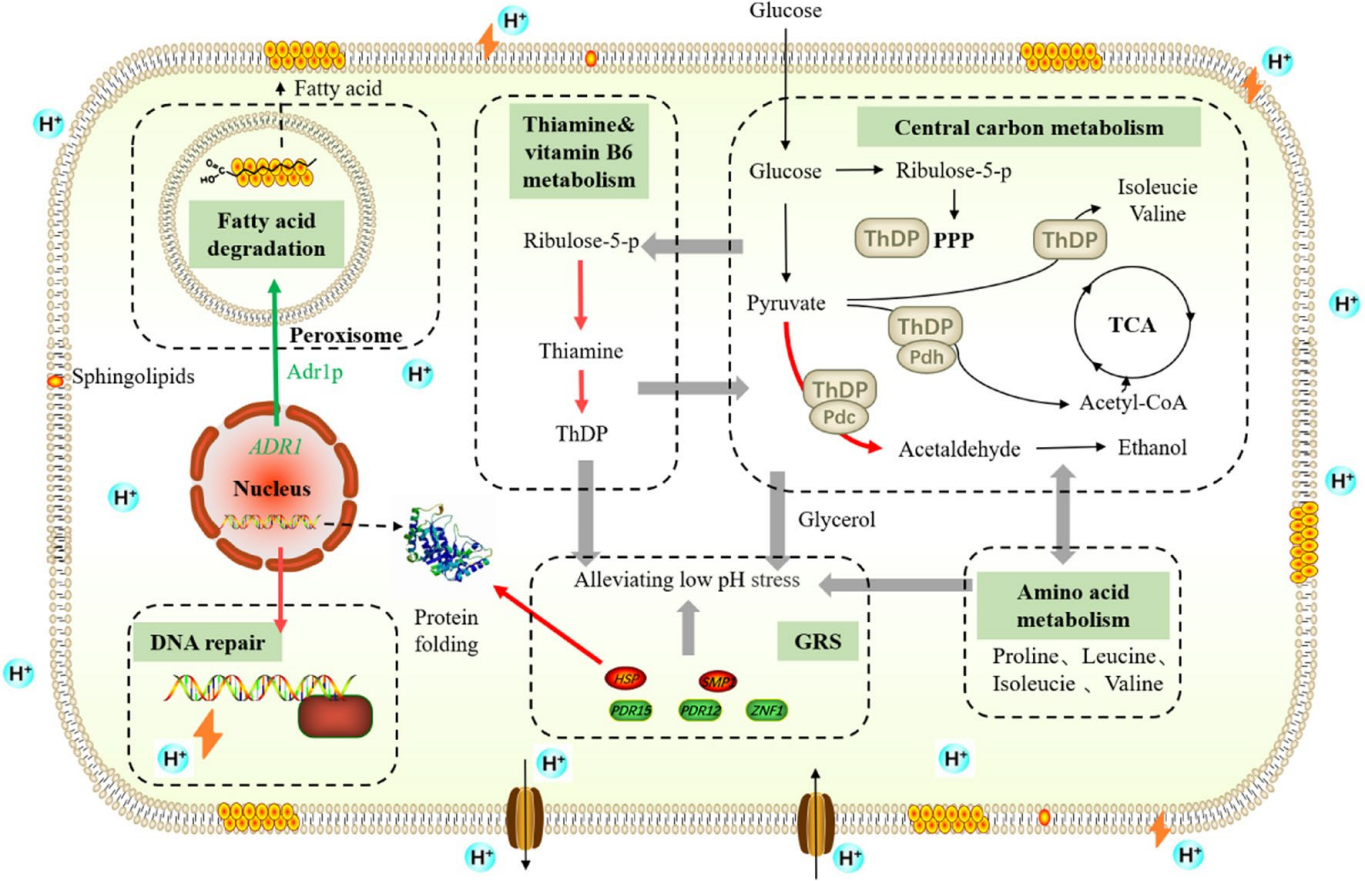
Schematic diagram of the low pH tolerance mechanism of haploid strains B3 and C3 under pH 2.5

Cell membrane provide a constant intracellular environment for cell growth and metabolism. Maintenance of proper membrane structure and function is a prerequisite for all cellular metabolic activities. Low pH usually leads to morphological changes in cells, which is a consequence of the damaged lipoidal cell membrane and decreased fluidity [36]. Some microbes regulate membrane fluidity by modulating fatty acid metabolism, such as modulating fatty acid composition, altering the unsaturation ratio and the isomerization of unsaturated fatty acids, and altering either the proportion or type of branched chain of fatty acids [36]. It has been reported that higher unsaturation ratios of membrane fatty acids contribute to cell survival at low pH [36]. In the present study, the downregulation of *POX1*, *POT1*, and *SPS19* involved in long-chain fatty acids degradation might be conducive to the accumulation of long-chain fatty acids and therefore regulate the fluidity of cell membrane under low pH condition. Consistent with this, carbon source-responsive transcriptional regulator Adr1p was also down-regulated at pH 2.5. Adr1p coordinates the biochemical pathways that generate acetyl-CoA and NADH from non-fermentable substrates [37]. In the present study, most DEGs involved in fatty acid metabolism, including *POT1*, *SPS19*, *TES1*, *POX1*, *EC11*, and *CTA1*, can be positively regulated by Adr1p based on the analysis using the YEASTRACT database. Adr1p might have an even more general function as a transcriptional activator under suboptimal growth conditions [38]. In addition, *STP4* was upregulated in B3, C3, and BC3. Stp4p (predicted transcription factor) might participate in the control of cell membrane homeostasis by sensing depletion of Sphingolipids (SLs), structural components of cell membranes. It translocates to the nucleus to induce the expression of genes involved in SLs synthesis [39]. The above DEGs might be jointly involved in maintaining cell membrane homeostasis under low pH condition (Fig. 9).

Maintaining chromosomal stability is an important aspect of stress tolerance. At pH 2.5, the DEGs involved in DNA repairment were upregulated (Table 3). *BUD16* and *THI4* are not only involved in thiamine synthesis. *BUD16* encodes yeast Pdxk, which is not only a critical enzyme in vitamin B6 metabolism but also in genome integrity maintenance via its role in maintaining adequate PLP [20]. Thi4p participates in both HET biosynthesis and maintenance of biological activity of mitochondrial DNA [40]. *RAD10* is required to repair HO endonuclease -induced double-strand breaks [41]. Hst3p and Hst4p prevent genome instability by tuning levels of both coding and noncoding gene transcription [42]. The expression of *YJR096W* was induced by methyl methanesulphonate (MMS), a kind of DNA-damaging agent [43]. The above DEGs might be involved in the co-maintenance of chromosome homeostasis under low pH condition.

The downregulation of DEGs involved in fatty acid degradation and the upregulation of DEGs involved in DNA repair was conducive to maintaining cell membrane homeostasis and chromosome homeostasis under low pH condition. The above two aspects of regulation might provide *S. cerevisiae* with a certain degree of low pH tolerance.

### Metabolic regulation of cells under low pH condition

The metabolism of Haploids B3 and C3 appeared to be strongly thiamine-dependent (Fig. 9). Haploids B3 and C3 might regulate their central carbon metabolism and amino acid metabolism through thiamine-dependent pathways, thereby their growth and fermentation ability under low pH condition were improved. The active form of thiamine is ThDP. ThDP participates in the ethanol production pathway, TCA cycle, PPP, and amino acid synthesis through ThDP-dependent enzymes pyruvate decarboxylase (Pdc), pyruvate dehydrogenase (Pdh), and transketolase (Tkl), α-acetolactate synthase, respectively [28, 30].

ThDP is particularly critical in the metabolism of pyruvate [28], the end product of glycolysis. In the ethanol production pathway, ThDP-dependent enzymes Pdc catalyzes the irreversible conversion of pyruvate to acetaldehyde [28], which is then converted into ethanol. However, ThDP is destroyed when bound to ThDP-dependent enzymes in the normal catalytic reaction [44]. Under pH 2.5, B3 and C3 might require more Pdc (*PDC5* was upregulated), which further led to the more consumption of ThDP. As a result, B3 and C3 should produce more ThDP to maintain normal metabolic activities, which in turn led to the upregulation of DEGs involving in the entire thiamine metabolic pathway. This regulation may be beneficial in promoting the production of ethanol under pH 2.5. Previous studies have shown that thiamine plays an important role in the ethanol fermentation of *S. cerevisiae*. Xu et al. found that the production of pyruvate and ethanol of *S. cerevisiae* could be controlled by regulating the thiamine synthesis [45]. Shi et al. found that overexpression of *THI4* increased the glucose consumption rate, ethanol concentration, and osmotic stress tolerance of yeast BY4741 [29]. Zeng et al. found that the upregulation of DEGs involved in thiamine synthesis possibly contributed to the improvement of xylose metabolism [27].

The expression of most genes encoding enzymes involved in ThDP synthesis and thiamine utilization was low or absent in the presence of exogenous thiamine, but they would be strongly upregulated when thiamine became limited [30]. In this study, under pH 2.5, the DEGs in the thiamine synthesis pathway, such as *THI2*, *SNZ2*//*3*, *SNO2*//*3*, etc., were upregulated to different degrees in haploids B3 and C3, suggesting that B3 and C3 need more thiamine to cope with low-pH stress. It was reported that thiamine strongly repressed the expression of *PDC5*, however, in the absence of external thiamine, the expression of *PDC5* is upregulated [28, 30]. In the present study, the significant upregulation of *PDC5* in B3 and C3, therefore, indicated that thiamine might be insufficient in B3 and C3. In low pH-tolerant strains, thiamine might participate in a broader metabolic regulation.

On the other hand, the downregulation of amino acid metabolism might also be related to intracellular thiamine deficiency. For example, the biosynthesis of isoleucine and valine, which stem from pyruvate, are also dependent on ThDP and there is evidence that the catabolism of branched-chain amino acids also proceeds via ThDP-catalyzed reactions [28].

### Stress regulation at low pH condition

The intermediate metabolites of thiamine, amino acid, and glycerol are related to GSR. Thiamine can alleviate the redox stress in *S. cerevisiae* [27, 46]. Some studies reported that thiamine affected cellular redox state through ThDP-dependent enzymes such as transketolase or α-ketoglutarate dehydrogenase [47, 48], or protected cellular redox balance by producing NADPH and glutathione [46]. By supplied with 1.5 µM thiamine, *Schizosaccharomyces pombe* Lindner liquefacient wild type 972 h showed the highest tolerance against oxidative stress [46]. When *S. pombe* wild type 972 h and *S. pombe* ird11 mutant were exposed to H_2_O_2_, the expression of genes (*THI2*, *THI3,* and *PHO1*) involved in thiamine biosynthesis and transport were upregulated even if thiamine was present in the medium [49]. In addition, thiamine also has a certain effect on the tolerance of thermal stress and osmotic stress of *S. cerevisiae*. Yeast BY4741 exhibited improved tolerance to oxidative stress (1 mM H_2_O_2_), osmotic stress (1 M sorbitol), and thermal stress (high temperature, 42 °C) when grown in medium supplemented with 1.4 μM thiamine [47].

As for amino acid metabolism, Nugroho et al. reported that the addition of proline protected the cells from acid stress (lactic acid) by combating acid-induced oxidative stress [50]. Li et al. also found that under acetic acid and formic acid stress, most DEGs involved in amino acid metabolism of *S. cerevisiae* S6 were down-regulated, and amino acid metabolism played important role in response to acetic and formic acids [51]. Changes in the metabolism of amino acids (proline, aspartic acid, etc.) also affect the tolerance of strains to oxidative stress [52], osmotic pressure, ethanol stress [53], and freezing [54]. For example, an appropriate level of proline protected yeast cells from damage caused by various stresses, including freezing, desiccation, osmolarity, oxidation, and ethanol [55]. The results of Ohta et al. showed that simultaneous deletion of *LEU4* and *LEU9* (leading to accumulation of valine) significantly enhanced ethanol tolerance of *S. cerevisiae* [56]. In the present study, the genes *PUT1* (encoding proline oxidase), *LEU1,* and *LEU4* were also downregulated.

The glycerol accumulation of low pH-tolerant strains was significantly increased at pH 2.5. Low pH will damage the cell wall, and the CWI pathway is the main mechanism for cell tolerance to low pH. Cell wall injury might mimic the effects of high osmotic shock and activates the HOG pathway [11]. The increase of glycerol concentration in the present study might also be a protective mechanism for cells at low pH condition.

In addition, genes involved in stress response differentially expressed under pH 2.5. The genes of heat shock proteins (Hsps) were mostly upregulated except for *HSP12*. Consistent with the results of De Melo et al., low pH activated GSR, which is mainly a heat shock response [3]. Hsps implicate growth-related processes such as DNA replication, cell division, transcription, translation, protein folding, membrane protein functions, and protein transport. They play important roles in the survival and maintenance of normal functions of cells under heat, oxidative stress, heavy metals, ethanol, or other toxic substances [57, 58]. Previous studies suggested that *S. cerevisiae* itself has cross-tolerance to different kinds of stress [59], for example, acid-tolerant strains also show heat tolerance [3, 9]. Under pH 2.5, yeast cells might quickly respond to the decrease of pH by inducing the expression of genes in GSR, which was consistent with previous studies [3, 11].

## Conclusion

In this study, comparative transcriptome analysis was carried out for the original strain KF-7, and two haploid strains B3 and C3 with improved low pH tolerance and their mating strain BC3 obtained by long-term continuous fermentation under low-pH stress, sporulation, and mating. The results found that the DEGs at pH 2.5 were involved in multiple metabolism pathways, among which the more variable pathways were fatty acid degradation, thiamine and vitamin B6 biosynthesis, amino acid metabolism, GSR and DNA repairment. The mechanisms of low pH tolerance of strains B3 and C3 could be summarized into three aspects: maintenance of cellular homeostasis, metabolic regulation, and stress regulation. Under low pH condition, B3 and C3 maintained cell membrane homeostasis and chromosomal homeostasis via regulating cell membrane fluidity and DNA repairment. They regulated carbon and amino acid metabolism via the ThDP-dependent metabolic regulation. The increased generation of intermediate metabolites including thiamine, glycerol, and amino acids, and the induced expression of genes in GSR synergistically provided yeast cells the tolerance to low pH condition.

## Materials and methods

### Strains and media

*Saccharomyces cerevisiae* flocculating strains of KF-7 [12], B3, C3 and BC3 were used in this study. Mutant strains of B3, C3 and BC3 were obtained from KF-7 [13]. A mutant strain K12 with improved low pH-tolerance was first obtained from KF-7 through 80 days-evolution based on continuous fermentation using a 1 L- continuous stirred tank reactor. In continuous fermentation, YPD150 medium (10 g/L yeast extract, 20 g/L peptone, 150 g/L glucose) was used. The pH of the fermentation broth was gradually reduced from 4.0 to 2.7 using an HCl solution during the 80 d-operation. When the fermentation was stable under pH 2.7, the mutant strains were isolated through single colony isolation, and strain K12 with the best acid tolerance was selected via growth screening under pH 2.7 and fermentation evaluation under pH 2.5. Strain K12 was then cultured on a sporulation agar plate (1 g/L yeast extract, 1 g/L potassium acetate, 0.5 g/L glucose, and 20 g/L agar) to obtain spores. Then asci were dissected using a Singer MSM300 System (Singer Instruments, Watchet, UK) after the treatment with snail enzyme. Haploid strains of B3 (*MAT*a) and C3 (*MAT*α) with superior growth ability under pH 2.7 and fermentation capacity under pH 2.5 were obtained. Strain BC3 is a diploid obtained by mating B3 and C3. Strains B3 and C3 have much better low pH tolerance than K12 and BC3.

A YPD-agar plate (10 g/L yeast extract, 20 g/L peptone, 20 g/L glucose, and 20 g/L agar) was used for cell activation. YPD50 medium (10 g/L yeast extract, 20 g/L peptone, and 50 g/L glucose) was used for pre-cultivation. YPD130 medium (10 g/L yeast extract, 20 g/L peptone, 130 g/L glucose, pH 2.5 or pH 4.5) was used for batch fermentation.

### Batch fermentation

Yeast strains were stocked in 15% glycerol stocks at −80 °C and recovered on YPD-agar plates (natural pH of 5.4) before characterization. A loopful of cells was inoculated into a cotton-plugged 500-mL flask with 100 mL of YPD50 medium (natural pH of 5.4) and cultivated for 16 h (160 rpm, 30 °C) in a shaker. Fresh cells were collected by centrifugation (8000 × g, 2 min), and inoculated into a cotton-plugged 300-mL flask with 100 mL of YPD130 medium. The initial cell density was adjusted to 2 × 10^7^ cell/mL. The pH of the YPD130 medium was adjusted to 2.5 or 4.5 using an HCl solution. The flasks were incubated in a thermostat water bath (35 °C). The broth was magnetically stirred at 200 rpm. The batch fermentation experiments were conducted with two biological repeats.

### Analytical methods

The fermentation broth was centrifuged at 8000 × g for 2 min. The precipitated cells were resuspended using 0.5 M EDTA solution. The cell density was determined by using the hemocytometer. The supernatant was filtered through a 0.22 μm membrane filter and used for the analysis of the concentrations of residual glucose, ethanol, and glycerol. Glucose was determined using high-performance liquid chromatography (HPLC) (LC-10AD VP, Shimadzu, Kyoto, Japan) equipped with a fluorescence detector (RF-10A_XL_). The column Shimpack ISA-07/S2504 (4 mm i.d. × 25 cm L) was used. Buffer A (0.1 M borate buffer, pH 8.0) and buffer B (0.4 M borate buffer, pH 9.0) were used at a flow rate of 0.6 mL/min with a gradient from 100% buffer A (0% buffer B) to 0% buffer A (100% buffer B) at a changing rate of 2%/min. The sample injection volume was 20 μL and the running time was 20 min [60]. Ethanol was measured by an internal standard method (2-propanol was used as the internal standard) using gas chromatography (GC 353B, GL Sciences, Tokyo, Japan) with a flame ionization detector (FID) and a TC-1 capillary column (0.25 mm i.d. × 60 mL; d.f.: 0.25 μm). The oven temperature was set at 50 ℃ and the injection and detector temperatures were set at 180 ℃. Helium was used as the carrier gas and H_2_ as the flaming gas. The sample injection volume was 0.5 μL and the running time was 9 min [60]. Glycerol was assayed using HPLC (SCL-10A VP, Shimadzu, Kyoto, Japan) equipped with an Aminex HPX-87H column (300 × 7.8 mm) (Bio-Rad, Hercules, USA) and a RID-10A refractive index detector (Shimadzu, Kyoto, Japan). The HPLC was operated at 35 ℃ using 5 mM H_2_SO_4_ as mobile phase at 0.6 mL/min. The sample injection volume was 50 μL and the running time was 15 min [61].

### RNA extraction

For transcriptional analysis, yeast cells were harvested at 4 h under pH 4.5 and 8 h under pH 2.5 during batch fermentation with YPD130 medium, respectively. Total RNA was extracted using the Takara Yeast RNAiso Kit (Dalian, China). RNA quality and concentration were measured by agarose gel electrophoresis and NanoDrop 2000/2000C (Thermo Scientific, Waltham, USA). A total of 16 RNA samples (two biological repeats of each strain under each pH condition) were subjected to followed microarray analysis.

### Microarray analysis and quantitative real-time PCR

Microarray analysis was performed using the 7G Affymetrix GeneChip^®^ Yeast Genome 2.0 Array. The isolated total RNA was cleaned up with the RNeasy Kit (Qiagen, Hilden, Germany); 100 ng of total RNA was used for complementary DNA (cDNA) synthesis and to produce biotin-tagged complementary RNA (cRNA) with the GeneChip IVT Labeling Kit (Affymetrix, Santa Clara, CA). A total of 15 μg of fragmented cRNA, together with control oligo B2 and eukaryotic hybridization controls, was hybridized to each GeneChip array at 45 °C for 16 h (Affymetrix GeneChipHybridization Oven 640) according to the manufacturer’s instructions. After hybridization, the GeneChip arrays were washed, stained with streptavidin phycoerythrinonan (SAPE) on an Affymetrix Fluidics Station 450, and then scanned with the Affymetrix GeneChip Scanner 3000 7G. The data extraction and analysis were carried out using the Affymetrix GeneChip Command Console Software. The microarray data can be accessed through GEO accession through GSE210964.

To identify altered gene expression, the averages of two biological duplicates were compared, and the genes filtered with a fold change (Sample B / Sample A) ≥ 2 or ≤ 0.5 were considered as differentially expressed genes (DEGs). A Venn diagram was used for Venn mapping to observe the quantitative distribution of the DEGs in each group. The gene function was annotated based on the *Saccharomyces* Genome Database (SGD). KEGG pathway analyses were performed on the DEGs, and those pathways with a *P* ≤ 0.05 and enrichment ratio ≥ 0.2 were considered to be significantly enriched. *P* value was calculated based on the hypergeometric distribution. The enrichment ratio of each KEGG term was the number of DEGs involved in each KEGG term to the number of total genes involved in each KEGG term. The DEGs were shown on the KEGG pathway map according to the KEGG database. The DEGs were used to search for transcription factors (TFs) that have been experimentally shown to regulate the expression of the genes from documented associations in the YEASTRACT database. The protein interaction was analyzed based on the Metascape.

The RNA samples were also used for the real-time RT-qPCR validation of genes with varying transcript abundance. The cDNA was reversely transcribed from total RNA using the Takara PrimeScript™ RT reagent kit with gDNA Eraser (Perfect Real Time) (Dalian, China). Six genes, *ADY2*, *ATO2*, *BTN2*, *ENO1*, *ENO2*, and *HSP30* were chosen to quantify their relative expression levels in all 16 RNA samples, and the primers used for RT-qPCR are listed in Additional file 1: Table S1. RT-qPCR was performed according to the manufacturer’s manual for Takara SYBR^®^ Premix Ex TaqTM II (Tli RNaseH Plus) (Dalian, China). The expression level of each gene was normalized using the *ACT1* expression level as a reference. The fold change was determined by the 2^−ΔΔCT^ method [62]. Each sample was run in triplicate on a 96-well plate, and each group was repeated three times. The value of RT-qPCR was presented as the mean of the triplicates.

## Supplementary Information

**Additional file 1: Table S1** Primers used for RT-qPCR. **Fig. S1** The cluster of the gene expression level of each strain. **Fig. S2** Validation of transcriptome data by RT-qPCR. The fold change means the ratio of the expression level of a specific gene in experimental group samples to that in control samples. The *ACT1 *expression level was used as a reference in RT-qPCR. Panel A, B, C indicated groups B3 vs KF-7, C3 vs KF-7, and BC3 vs KF-7 under pH 4.5, while panel D, E, F indicated the conditions under pH 2.5, respectively.

## Data Availability

The datasets generated during the current study are available in the NCBI repository, https://www.ncbi.nlm.nih.gov/geo/query/acc.cgi?acc=GSE210964. The microarray data can be accessed through the GEO accession GSE210964.

## Abbreviations

*S. cerevisiae*: *Saccharomyces cerevisiae*
GSR: General stress response
PKA: Protein kinase A
PKC: Protein kinase C
CWI: Cell wall integrity
HOG: High osmolarity glycerol
ATP: Adenosine-triphosphate
DEGs: Differentially expressed genes
KEGG: Kyoto Encyclopedia of Genes and Genomes
Acetyl-CoA: Acetyl coenzyme A
ThDP: Thiamine and thiamine diphosphate
PN: Pyridoxine
PL: Pyridoxal
PM: Pyridoxamine
PLP: Pyridoxal 5′-phosphate
Pdxk: Pyridoxal kinase
PLPC: Pyridoxal 5′-phosphate synthase complex
HMP: 4-Amino-2-methyl-5-hydroxymethylpyrimidine
HET: 4-Methyl-5-β-hydroxyethylthiazole
ThP: Thiamin phosphate
MAPK: Mitogen-activated protein kinase
SLs: Sphingolipids
MMS: Methyl methanesulphonate
TCA cycle: Tricarboxylic acid cycle
PPP: Pentose phosphate pathway
Pdc: Pyruvate decarboxylase
Pdh: Pyruvate dehydrogenase
Tkl: Transketolase
NADPH: Nicotinamide adenine dinucleotide phosphate
Hsps: Heat-shock proteins
HPLC: High-performance liquid chromatography
FID: Flame ionization detector
DNA: Deoxyribonucleic acid
RNA: Ribonucleic Acid
cDNA: Complementary DNA
cRNA: Complementary RNA
SAPE: Streptavidin phycoerythrinonan
SGD: *Saccharomyces* Genome Database
TFs: Transcription factors
RT-qPCR: Quantitative real-time Polymerase Chain Reaction

## References

[1] Johnston NR, Nallur S, Gordon PB, Smith KD, Strobel SA (2020). Genome-wide identification of genes involved in general acid stress and fluoride toxicity in *Saccharomyces cerevisiae*. Front Microbiol.

[2] Lucena RM, Dolz-Edo L, Brul S, de Morais MA, Smits G (2020). Extreme low cytosolic pH is a signal for cell survival in acid stressed yeast. Genes (Basel).

[3] De Melo HF, Bonini BM, Thevelein J, Simoes DA, Morais MA (2010). Physiological and molecular analysis of the stress response of *Saccharomyces cerevisiae* imposed by strong inorganic acid with implication to industrial fermentations. J Appl Microbiol.

[4] Fletcher E, Feizi A, Bisschops MMM, Hallstrom BM, Khoomrung S, Siewers V, Nielsen J (2017). Evolutionary engineering reveals divergent paths when yeast is adapted to different acidic environments. Metab Eng.

[5] Kadar Z, Maltha SF, Szengyel Z, Reczey K, De Laat W (2007). Ethanol fermentation of various pretreated and hydrolyzed substrates at low initial pH. Appl Biochem Biotech.

[6] Brodeur G, Yau E, Badal K, Collier J, Ramachandran KB, Ramakrishnan S (2011). Chemical and physicochemical pretreatment of lignocellulosic biomass: a review. Enzyme Res.

[7] De Lucena RM, Elsztein C, Pita WD, de Souza RB, Paiva SDL, de Morais MA (2015). Transcriptomic response of *Saccharomyces cerevisiae* for its adaptation to sulphuric acid-induced stress. Anton Leeuw Int J G.

[8] Mitsumasu K, Liu ZS, Tang YQ, Akamatsu T, Taguchi H, Kida K (2014). Development of industrial yeast strain with improved acid- and thermo-tolerance through evolution under continuous fermentation conditions followed by haploidization and mating. J Biosci Bioeng.

[9] Benjaphokee S, Hasegawa D, Yokota D, Asvarak T, Auesukaree C, Sugiyama M, Kaneko Y, Boonchird C, Harashima S (2012). Highly efficient bioethanol production by a *Saccharomyces cerevisiae* strain with multiple stress tolerance to high temperature, acid and ethanol. N Biotechnol.

[10] Matsushika A, Suzuki T, Goshima T, Hoshino T (2017). Evaluation of *Saccharomyces cerevisiae GAS1* with respect to its involvement in tolerance to low pH and salt stress. J Biosci Bioeng.

[11] De Lucena RM, Elsztein C, Simoes DA, de Morais MA (2012). Participation of CWI, HOG and Calcineurin pathways in the tolerance of *Saccharomyces cerevisiae* to low pH by inorganic acid. J Appl Microbiol.

[12] Kida K, Kume K, Morimura S, Sonoda Y (1992). Repeated-batch fermentation process using a thermotolerant flocculating yeast constructed by protoplast fusion. J Ferment Bioeng.

[13] Luo Z, Tang YQ, Sun ZY, Kida K (2014). Breeding acid-tolerant *Saccharomyces cerevisiae* strain based on continuous ethanol fermentation under acid stressed condition. J Sichuan Univ: Nat Sci Ed.

[14] Ortiz-Muniz B, Carvajal-Zarrabal O, Torrestiana-Sanchez B, Aguilar-Uscanga MG (2010). Kinetic study on ethanol production using *Saccharomyces cerevisiae* ITV-01 yeast isolated from sugar cane molasses. J Chem Technol Biotechnol.

[15] Manzanares-Estreder S, Espi-Bardisa J, Alarcon B, Pascual-Ahuir A, Proft M (2017). Multilayered control of peroxisomal activity upon salt stress in *Saccharomyces cerevisiae*. Mol Microbiol.

[16] Swiegers JH, Dippenaar N, Pretorius IS, Bauer FF (2001). Carnitine-dependent metabolic activities in *Saccharomyces cerevisiae*: three carnitine acetyltransferases are essential in a carnitine-dependent strain. Yeast.

[17] Hiltunen JK, Mursula AM, Rottensteiner H, Wierenga RK, Kastaniotis AJ, Gurvitz A (2003). The biochemistry of peroxisomal beta-oxidation in the yeast *Saccharomyces cerevisiae*. FEMS Microbiol Rev.

[18] Stolz J, Vielreicher M (2003). Tpn1p, the plasma membrane vitamin B-6 transporter of *Saccharomyces cerevisiae*. J Biol Chem.

[19] Stolz J, Wohrmann HJP, Vogl C (2005). Amiloride uptake and toxicity in fission yeast are caused by the pyridoxine transporter encoded by bsu1(+) (car1(+)). Eukaryot Cell.

[20] Kanellis P, Gagliardi M, Banath JP, Szilard RK, Nakada S, Galicia S, Sweeney FD, Cabelof DC, Olive PL, Durocher D (2007). A screen for suppressors of gross chromosomal rearrangements identifies a conserved role for PLP in preventing DNA lesions. PLoS Genet.

[21] Paxhia MD, Downs DM (2019). *SNZ3* encodes a PLP synthase involved in thiamine synthesis in *Saccharomyces cerevisiae*. G3-Genes Genom Genet..

[22] Rodriguez-Navarro S, Llorente B, Rodriguez-Manzaneque MT, Ramne A, Uber G, Marchesan D, Dujon B, Herrero E, Sunnerhagen P, Perez-Ortin JE (2002). Functional analysis of yeast gene families involved in metabolism of vitamins B1 and B6. Yeast.

[23] Nishizawa M, Komai T, Morohashi N, Shimizu M, Toh-e A (2008). Transcriptional repression by the Pho4 transcription factor controls the timing of *SNZ1* expression. Eukaryot Cell.

[24] Argueso JL, Carazzolle MF, Mieczkowski PA, Duarte FM, Netto OVC, Missawa SK, Galzerani F, Costa GGL, Vidal RO, Noronha MF, Dominska M, Andrietta MGS, Andrietta SR, Cunha AF, Gomes LH, Tavares FCA, Alcarde AR, Dietrich FS, McCusker JH, Petes TD, Pereira GAG (2009). Genome structure of a *Saccharomyces cerevisiae* strain widely used in bioethanol production. Genome Res.

[25] Stambuk BU, Dunn B, Alves SL, Duval EH, Sherlock G (2009). Industrial fuel ethanol yeasts contain adaptive copy number changes in genes involved in vitamin B1 and B6 biosynthesis. Genome Res.

[26] McIlwain SJ, Peris D, Sardi M, Moskvin OV, Zhan FJ, Myers KS, Riley NM, Buzzell A, Parreiras LS, Ong IM, Landick R, Coon JJ, Gasch AP, Sato TK, Hittinger CT (2016). Genome sequence and analysis of a stress-tolerant, wild-derived strain of *Saccharomyces cerevisiae* used in biofuels research. G3-Genes Genom Genet.

[27] Zeng WY, Tang YQ, Gou M, Sun ZY, Xia ZY, Kida K (2017). Comparative transcriptomes reveal novel evolutionary strategies adopted by *Saccharomyces cerevisiae* with improved xylose utilization capability. Appl Microbiol Biotechnol.

[28] Hohmann S, Meacock PA (1998). Thiamin metabolism and thiamin diphosphate-dependent enzymes in the yeast *Saccharomyces cerevisiae*: genetic regulation. Biochim Biophys Acta.

[29] Shi XC, Zou YN, Chen Y, Ying HJ (2018). Overexpression of *THI4* and *HAP4* improves glucose metabolism and ethanol production in *Saccharomyces cerevisiae*. Front Microbiol.

[30] Mojzita D, Hohmann S (2006). Pdc2 coordinates expression of the *THI* regulon in the yeast *Saccharomyces cerevisiae*. Mol Genet Genom.

[31] Nosaka K, Onozuka M, Konno H, Kawasaki Y, Nishimura H, Sano M, Akaji K (2005). Genetic regulation mediated by thiamin pyrophosphate-binding motif in *Saccharomyces cerevisiae*. Mol Microbiol.

[32] Singleton CK (1997). Identification and characterization of the thiamine transporter gene of *Saccharomyces cerevisiae*. Gene.

[33] De Nadal E, Casadome L, Posas F (2003). Targeting the MEF2-like transcription factor Smp1 by the stress-activated Hog1 mitogen-activated protein kinase. Mol Cell Biol.

[34] Tangsombatvichit P, Semkiv MV, Sibirny AA, Jensen LT, Ratanakhanokchai K, Soontorngun N (2015). Zinc cluster protein Znf1, a novel transcription factor of non-fermentative metabolism in *Saccharomyces cerevisiae*. FEMS Yeast Res.

[35] Schuller C, Mamnun YM, Mollapour M, Krapf G, Schuster M, Bauer BE, Piper PW, Kuchler K (2004). Global phenotypic analysis and transcriptional profiling defines the weak acid stress response regulon in *Saccharomyces cerevisiae*. Mol Biol Cell.

[36] Guan NZ, Liu L (2020). Microbial response to acid stress: mechanisms and applications. Appl Microbiol Biotechnol.

[37] Young ET, Dombek KM, Tachibana C, Ideker T (2003). Multiple pathways are co-regulated by the protein kinase Snf1 and the transcription factors Adr1 and Cat8. J Biol Chem.

[38] Simon M, Adam G, Rapatz W, Spevak W, Ruis H (1991). The *Saccharomyces cerevisiae ADR1* gene is a positive regulator of transcription of genes encoding peroxisomal proteins. Mol Cell Biol.

[39] Lebesgue N, Megyeri M, Cristobal A, Scholten A, Chuartzman SG, Voichek Y, Scheltema RA, Mohammed S, Futerman AH, Schuldiner M, Heck AJR, Lemeer S (2017). Combining deep sequencing, proteomics, phosphoproteomics, and functional screens to discover novel regulators of sphingolipid homeostasis. J Proteome Res.

[40] Machado CR, Praekelt UM, de Oliveira RC, Barbosa ACC, Byrne KL, Meacock PA, Menck CFM (1997). Dual role for the yeast *THI4* gene in thiamine biosynthesis and DNA damage tolerance. J Mol Biol.

[41] Ivanov EL, Haber JE (1995). *RAD1* and *RAD10*, but not other excision repair genes, are required for double-strand break-induced recombination in *Saccharomyces cerevisiae*. Mol Cell Biol.

[42] Feldman JL, Peterson CL (2019). Yeast sirtuin family members maintain transcription homeostasis to ensure genome stability. Cell Rep.

[43] Lee MW, Kim BJ, Choi HK, Ryu MJ, Kim SB, Kang KM, Cho EJ, Youn HD, Huh WK, Kim ST (2007). Global protein expression profiling of budding yeast in response to DNA damage. Yeast.

[44] McCourt JA, Nixon PF, Duggleby RG (2006). Thiamin nutrition and catalysis-induced instability of thiamin diphosphate. Br J Nutr.

[45] Xu GQ, Hua Q, Duan NJ, Liu LM, Chen J (2012). Regulation of thiamine synthesis in *Saccharomyces cerevisiae* for improved pyruvate production. Yeast.

[46] Kartal B, Palabiyik B (2019). Thiamine leads to oxidative stress resistance via regulation of the glucose metabolism. Cell Mol Biol.

[47] Wolak N, Kowalska E, Kozik A, Rapala-Kozik M (2014). Thiamine increases the resistance of baker's yeast *Saccharomyces cerevisiae* against oxidative, osmotic and thermal stress, through mechanisms partly independent of thiamine diphosphate-bound enzymes. FEMS Yeast Res.

[48] Depeint F, Bruce WR, Shangari N, Mehta R, O'Brien PJ (2006). Mitochondrial function and toxicity: role of the B vitamin family on mitochondrial energy metabolism. Chem Biol Interact.

[49] Kartal B, Akcay A, Palabiyik B (2018). Oxidative stress upregulates the transcription of genes involved in thiamine metabolism. Turk J Biol.

[50] Nugroho RH, Yoshikawa K, Shimizu H (2015). Metabolomic analysis of acid stress response in *Saccharomyces cerevisiae*. J Biosci Bioeng.

[51] Li B, Xie CY, Yang BX, Gou M, Xia ZY, Sun ZY, Tang YQ (2020). The response mechanisms of industrial *Saccharomyces cerevisiae* to acetic acid and formic acid during mixed glucose and xylose fermentation. Process Biochem.

[52] Krems B, Charizanis C, Entian KD (1996). The response regulator-like protein Pos9/Skn7 of *Saccharomyces cerevisiae* is involved in oxidative stress resistance. Curr Genet.

[53] Ming M, Wang XY, Lian LL, Zhang H, Gao WX, Zhu B, Lou DW (2019). Metabolic responses of *Saccharomyces cerevisiae* to ethanol stress using gas chromatography-mass spectrometry. Mol Omics.

[54] Morita Y, Nakamori S, Takagi H (2002). Effect of proline and arginine metabolism on freezing stress of *Saccharomyces cerevisiae*. J Biosci Bioeng.

[55] Takagi H (2008). Proline as a stress protectant in yeast: physiological functions, metabolic regulations, and biotechnological applications. Appl Microbiol Biotechnol.

[56] Ohta E, Nakayama Y, Mukai Y, Bamba T, Fukusaki E (2016). Metabolomic approach for improving ethanol stress tolerance in *Saccharomyces cerevisiae*. J Biosci Bioeng.

[57] Miura T, Minegishi H, Usami R, Abe F (2006). Systematic analysis of *HSP* gene expression and effects on cell growth and survival at high hydrostatic pressure in *Saccharomyces cerevisiae*. Extremophiles.

[58] Richter K, Haslbeck M, Buchner J (2010). The heat shock response: life on the verge of death. Mol Cell.

[59] Berry DB, Gasch AP (2008). Stress-activated genomic expression changes serve a preparative role for impending stress in yeast. Mol Biol Cell.

[60] Tang YQ, An M, Liu K, Nagai S, Shigematsu T, Morimura S, Kida K (2006). Ethanol production from acid hydrolysate of wood biomass using the flocculating yeast *Saccharomyces cerevisiae* strain KF-7. Process Biochem.

[61] Li YC, Gou ZX, Liu ZS, Tang YQ, Akamatsu T, Kida K (2014). Synergistic effects of *TAL1* over-expression and *PHO13* deletion on the weak acid inhibition of xylose fermentation by industrial *Saccharomyces cerevisiae* strain. Biotechnol Lett.

[62] Livak KJ, Schmittgen TD (2001). Analysis of relative gene expression data using real-time quantitative PCR and the 2(T)(-Delta Delta C) method. Methods.

