# Supplementary material for: Saccharomyces cerevisiae employs complex regulation strategies to tolerate low pH stress during ethanol production
Source: Microb Cell Fact. 2022 Nov 24;21:247. doi: 10.1186/s12934-022-01974-3 (PMC9685915; doi:10.1186/s12934-022-01974-3)

*Saccharomyces cerevisiae* employs complex regulation strategies to tolerate low pH stress during ethanol production

Yajing Wu, Bo Li, Bu Miao*, Caiyun Xie*, Yue-Qin Tang

**Additional file 1 - content**

**Table S1** Primers used for RT-qPCR.

**Fig. S1** The cluster of the gene expression level of each strain

**Fig. S2** Validation of transcriptome data by RT-qPCR. The fold change means the ratio of the expression level of a specific gene in experimental group samples to that in control samples. The *ACT1* expression level was used as a reference in RT-qPCR. Panel A, B, C indicated groups B3 vs KF-7, C3 vs KF-7, and BC3 vs KF-7 under pH 4.5, while panel D, E, F indicated the conditions under pH 2.5, respectively.

**Table S1** Primers used for RT-qPCR.

| Target gene | Primer | Sequence (5’~3’) |
| --- | --- | --- |
| *ACT1* | Rt-ACT1 F | ATGCAAACCGCTGCTCAA |
|  | Rt-ACT1 R | AGTTTGGTCAATACCGGCAGA |
| *ADY2* | Rt-ADY2 F | CCTTCGCGTTGACGACATT |
|  | Rt-ADY2 R | ACCAAACCACCATAAAACATAGCAC |
| *ATO2* | Rt-ATO2 F | CGCAAATCCTGCTCCACTA |
|  | Rt-ATO2 R | GCACACCCAACAACAACATT |
| *BTN2* | Rt-BTN2 F | CGGAGAAAGCGAAAGAACCA |
|  | Rt-BTN2 R | TGGCAGCTTTTTCCTGTTCTG |
| *ENO1* | Rt-ENO1 F | GCTTTCGTTAAGGCTAACATTG |
|  | Rt-ENO1 R | AAGAAACACCCAAGATAGCG |
| *ENO2* | Rt-ENO2 F | CGCTATCTTGGGTGTCTCCA |
|  | Rt-ENO2 R | GCACCAGTTGGAGCAATCAT |
| *HSP30* | Rt-HSP30 F | TTCAACCAGACGGTGAGGCTA |
|  | Rt-HSP30 R | CCTTGGCAATTTGCCATCA |

**Fig. S1**


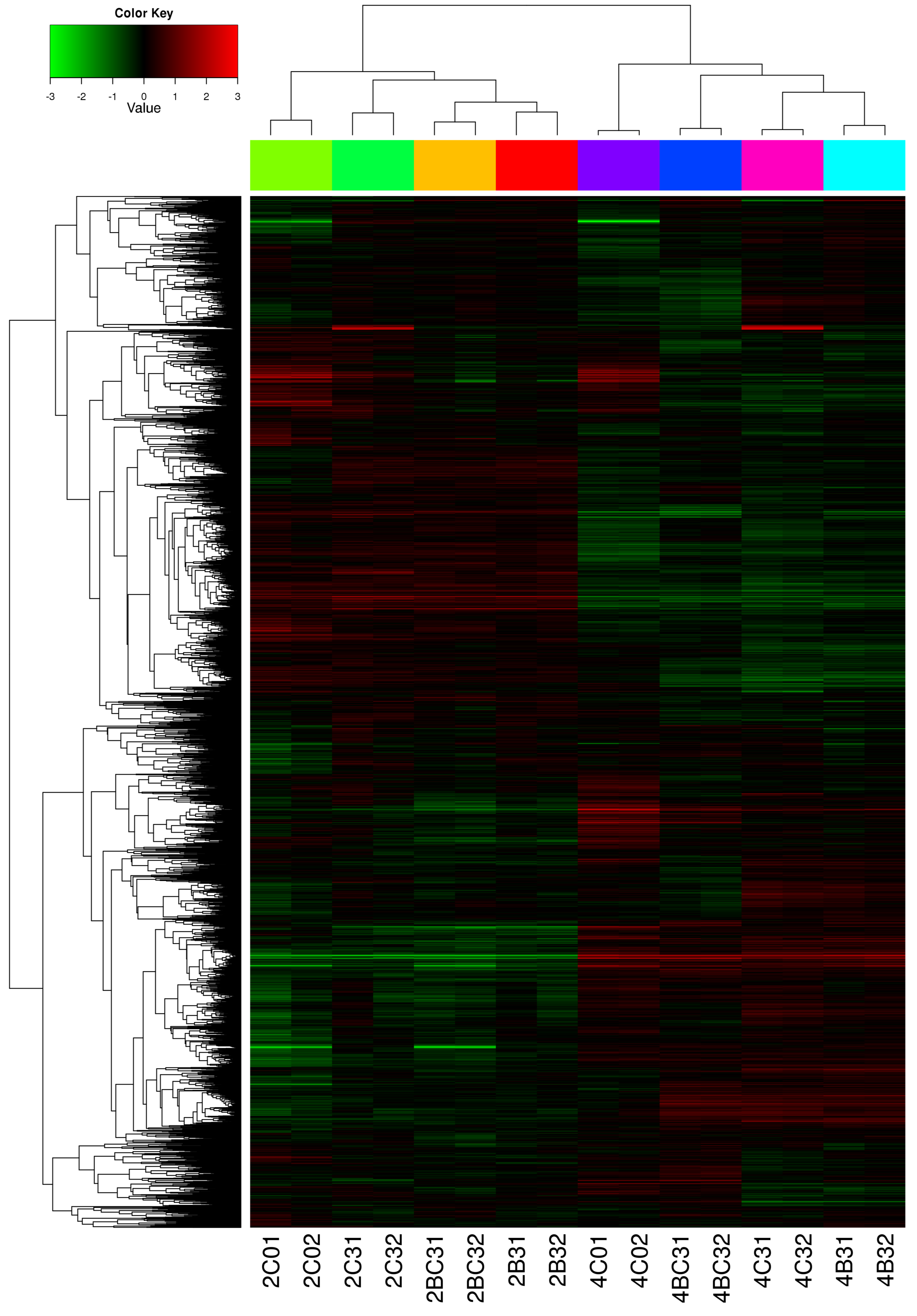


**Fig. S2**


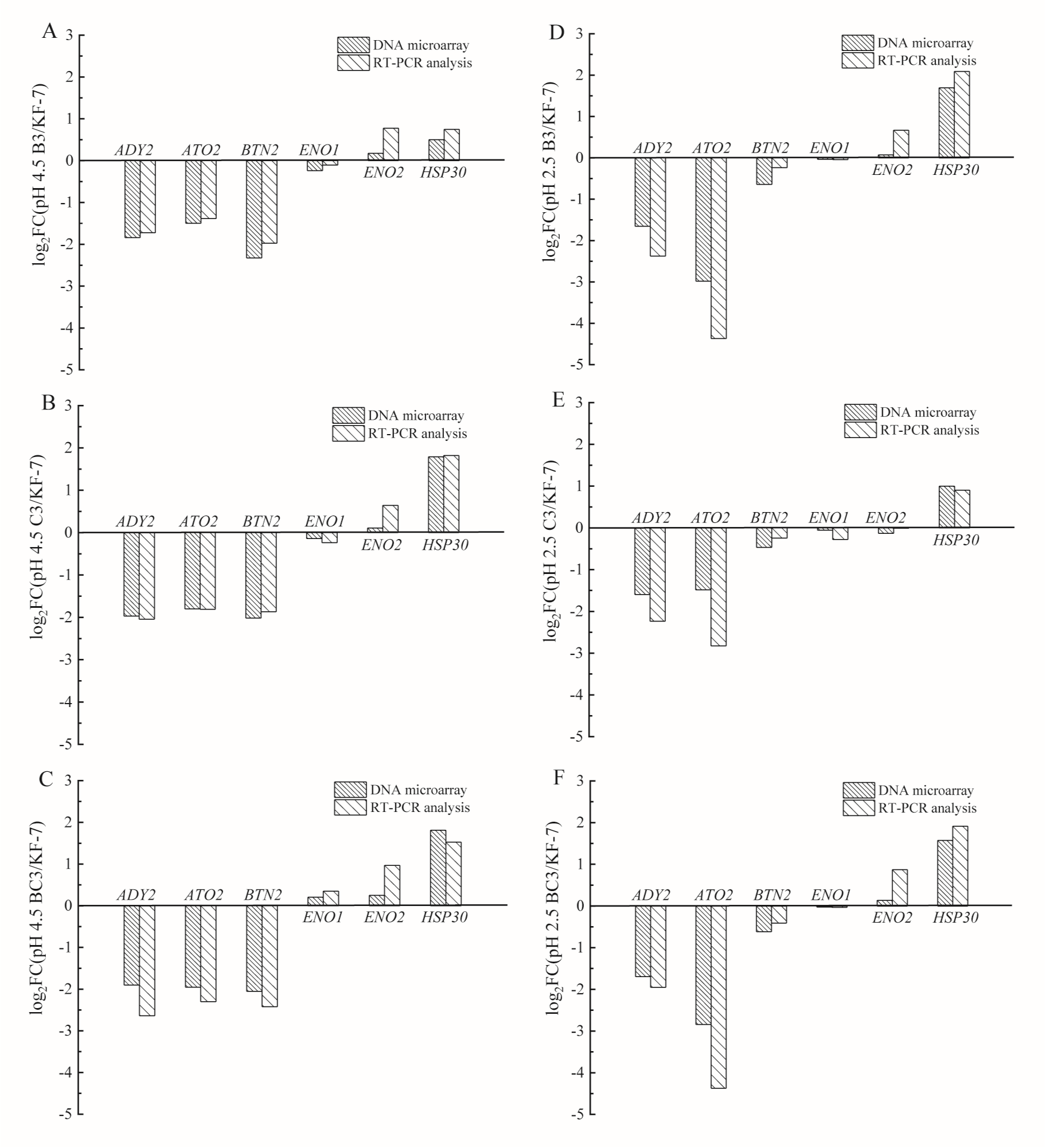

Supplement: Supplementary file 1 — Additional file 1: Table S1 Primers used for RT-qPCR. Fig. S1 The cluster of the gene expression level of each strain. Fig. S2 Validation of transcriptome data by RT-qPCR. The fold change means the ratio of the expression level of a specific gene in experimental group samples to that in control samples. The ACT1 expression level was used as a reference in RT-qPCR. Panel A, B, C indicated groups B3 vs KF-7, C3 vs KF-7, and BC3 vs KF-7 under pH 4.5, while panel D, E, F indicated the conditions under pH 2.5, respectively. [file 12934_2022_1974_MOESM1_ESM.docx]
